# Intestinal Obstruction for Anisakiasis: Surgical and Physical Therapy Treatment

**DOI:** 10.3390/jcm12134470

**Published:** 2023-07-04

**Authors:** Fernando Cózar-Bernal, Jorge Góngora-Rodríguez, Carmen Ayala-Martínez, Francisco Javier Martín-Vega, Maria Jesus Vinolo-Gil, Manuel Rodríguez-Huguet

**Affiliations:** 1 Servicio de Cirugía Torácica, Hospital Universitario Virgen Macarena, 41009 Sevilla, Spain; fcozarbernal@gmail.com; 2 Department of Nursing and Physiotherapy, University of Cádiz, 11009 Cádiz, Spain; javier.martin@uca.es (F.J.M.-V.); mariajesus.vinolo@uca.es (M.J.V.-G.); manuel.rodriguez@uca.es (M.R.-H.); 3 Department of Physiotherapy, Osuna School University, University of Sevilla, 41640 Sevilla, Spain; 4 Doctoral School, University of Cádiz, 11003 Cádiz, Spain; carmenmaria.ayamar@alum.uca.es; 5 Rehabilitation Clinical Management Unit, Interlevels-Intercenters Hospital Puerta del Mar, Hospital Puerto Real, Cadiz Bay-La Janda Health District, 11006 Cadiz, Spain; 6 Biomedical Research and Innovation Institute of Cadiz (INiBICA), Research Unit, Puerta del Mar University Hospital, University of Cadiz, 11009 Cadiz, Spain

**Keywords:** anisakiasis, intestinal obstruction syndrome, negative pressure, physical therapy modalities, zoonosis

## Abstract

Anisakiasis, a zoonotic disease that can lead to small intestine obstruction, has seen a significant rise in Spain. The country has become the first in Europe with an annual incidence of 8000 cases, primarily due to the popularity of consuming exotic dishes of undercooked or raw fish and the impact of climate change. The clinical presentation of anisakiasis can mimic symptoms of acute appendicitis or intestinal obstruction, leading to potential misdiagnosis. This case report describes a 37-year-old patient with no significant medical history who presented abdominal distension and intense pain in the right lower quadrant. The patient underwent surgery and received physiotherapy treatment, including therapeutic exercises and pulsed-pressure myofascial vacuum therapy, to facilitate functional recovery. The increasing incidence of anisakiasis in Spain underscores the need to consider it in the differential diagnosis of digestive diseases, given the high consumption of poorly prepared or raw fish in the region.

## 1. Introduction

Zoonosis is an infectious disease that can be transmitted between different species, including from animals to humans. Parasites are one of the causative agents of these diseases, and animals can act as vectors for their transmission. Fish, for instance, have been identified as one of the animal groups that can transmit parasites to humans [1]. However, according to Ziarati et al. (2022) [1], the detection of aquatic zoonotic cases is relatively lower compared to zoonotic diseases originating from other animals. This lower detection rate could potentially be attributed to inadequate monitoring and surveillance measures.

Furthermore, it is important to note that even though there may be a lower number of diagnosed cases, it does not diminish the potential severity and potential for fatal consequences. It is worth mentioning that, particularly in cases of parasitic nematodes, long-term control heavily relies on chemotherapeutic treatments. However, the prolonged use or incorrect dosage of these treatments can lead to the development of drug resistance [2,3]. Despite these challenges, researchers continue to isolate and analyze compounds derived from plants that possess nematicidal properties. This ongoing effort aims to discover effective alternatives for controlling nematode infections [3,4]. 

Anisakiasis, also known as anisakiosis, refers to the parasitic infection of humans exclusively caused by the third-stage larvae of the Anisakis simplex sensu lato parasite. It is important to distinguish anisakiasis from anisakidosis, which encompasses diseases caused by various members of the Anisakidae family [1]. The larvae of Anisakis have been found in raw or frozen fish fillets, including cod fillets and fish fingers [5]. However, the risk of encountering these nematodes appears to be insignificant, especially in fish sourced from aquaculture farms [6].

Anisakiasis is primarily contracted through the consumption of raw or undercooked fish that is infested with the larvae of this nematode [7,8]. According to Ziarati et al. (2022) [1], the increasing popularity of exotic dishes featuring raw or undercooked fish, coupled with climate change, are considered the main contributing factors to the rise in zoonotic health issues caused by fish. In Spain, where fish consumption is high and culinary trends lean towards undercooked or raw preparations, the incidence of anisakiasis has significantly increased in recent years [9]. In fact, Spain has the highest reported incidence of anisakiasis among all European countries, with approximately 8000 cases annually [9]. It is worth noting that between 25% and 80% of fish found in markets may be infested with Anisakis, with certain species, such as hake, having potentially higher rates of infestation [9]. Japan, renowned for its exquisite cuisine featuring raw or semi-raw fish, also experiences a high prevalence of anisakiasis [10]. These findings highlight the importance of proper cooking and food safety practices, particularly when consuming fish that may be at risk of Anisakis infestation.

After the fish has died, the nematode larvae ensure their survival by migrating from the fish’s gastrointestinal tract to its muscle tissue and nearby organs [3]. This underscores the importance of implementing rigorous controls on fish prior to its consumption in raw or undercooked form. Prevention measures aim to deactivate and eliminate this parasite through freezing or thermal procedures. However, it should be noted that the freezing and subsequent thawing process can lead to the deterioration of the fish, causing the meat to become tender due to the drip resulting from the storage of ice crystals formed during freezing [3]. Currently, ongoing research is focused on exploring alternative processing techniques for prevention. These include the potential use of ozone, microwaves, ionizing radiation, ohmic heating, high hydrostatic pressure, and chemical products [9]. These methods are being investigated to determine their effectiveness in preventing anisakiasis while minimizing negative impacts on fish quality.

Among the various methods employed for parasite inactivation, it is noteworthy to mention the use of pulsed electric fields, which have been widely utilized as a non-thermal approach to inactivate microorganisms present in liquid foods [11]. This technique involves applying high-intensity electric fields through short-duration pulses intermittently without raising the temperature of the meat [11]. The process entails placing two electrodes within the product submerged in an aqueous solution to facilitate the passage of electric current. Research conducted on the application of pulsed electric fields has shown promising results and suggests that it could serve as an alternative to the freezing process for parasite inactivation while preserving the quality of the fish meat [9]. This method offers the potential to inactivate parasites effectively without compromising the sensory attributes of the fish. 

The symptoms are caused by two pathophysiological mechanisms: IGE-mediated immediate hypersensitivity allergic reaction and gastrointestinal inflammatory reaction. There are two clinical presentations based on the extent of penetration into the mucous membrane: the non-invasive luminal form (the parasite resides in the intestinal mucosa and is typically asymptomatic. It is expelled through feces or vomit) and the invasive gastrointestinal form (the parasite penetrates the submucosal layer), which is the more prevalent. The invasive form can be further classified into two distinct clinical presentations: gastroduodenal (65%) and intestinal (35%, with the terminal ileum being the most frequently affected site) [12].

The clinical manifestation of the intestinal form can sometimes lead to misleading suspicions of acute appendicitis or intestinal obstruction [13]. Intestinal obstruction is a syndrome that accounts for approximately 20% of emergency surgeries in hospitals. It is characterized by the blockage of gases and feces in any segment of the intestine, which can vary in terms of the degree of occlusion (complete or incomplete) and the duration of the obstruction [14].

## 2. Case Description

We present the case of a 37-year-old patient with no significant medical history who experienced abdominal distension for several days. In the last two days, the patient also developed acute and severe abdominal pain localized to the right lower quadrant without fever or vomiting. The patient lost consciousness; as a result, the patient sought medical attention at the emergency department, and emergency diagnostic tests were performed.

During the abdominal examination, the abdomen was found to be soft and depressible. Superficial and deep palpation in the mesogastrium and right lower quadrant elicited pain. No signs of peritoneal irritation were observed. The laboratory analysis revealed only an elevated C-reactive protein level of 81 mg/L (this is an indicator of mortality in cases of abdominal sepsis [15], normal values are around 5 mg/L [16]), while other parameters were within the normal range.

To rule out appendicitis, an abdominal ultrasound was performed. The appendix appeared normal, with no signs of inflammation. However, the distal ileum loops were dilated with a diameter greater than 3 cm, accompanied by reduced peristalsis and the presence of liquid content. Additionally, a minimal amount of free peritoneal fluid was detected in the right lower quadrant [17].

**Figure 1:**
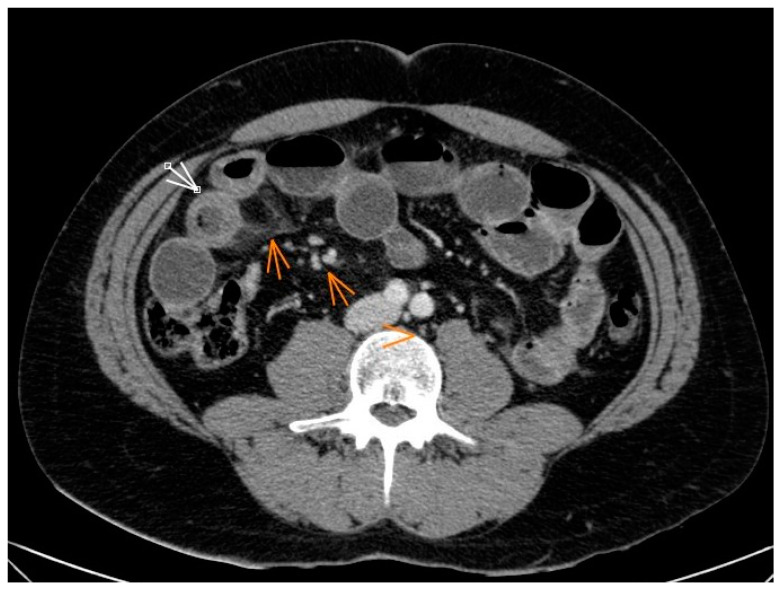
CT abdomen: Thickening of the wall of a short segment of the distal ileum with submucosal edema. Infiltration of mesenteric fat and adjacent subcentimeter lymph nodes. The arrows represent asymmetric mural thickening of the intestinal wall with submucosal edema.

**Figure 2:**
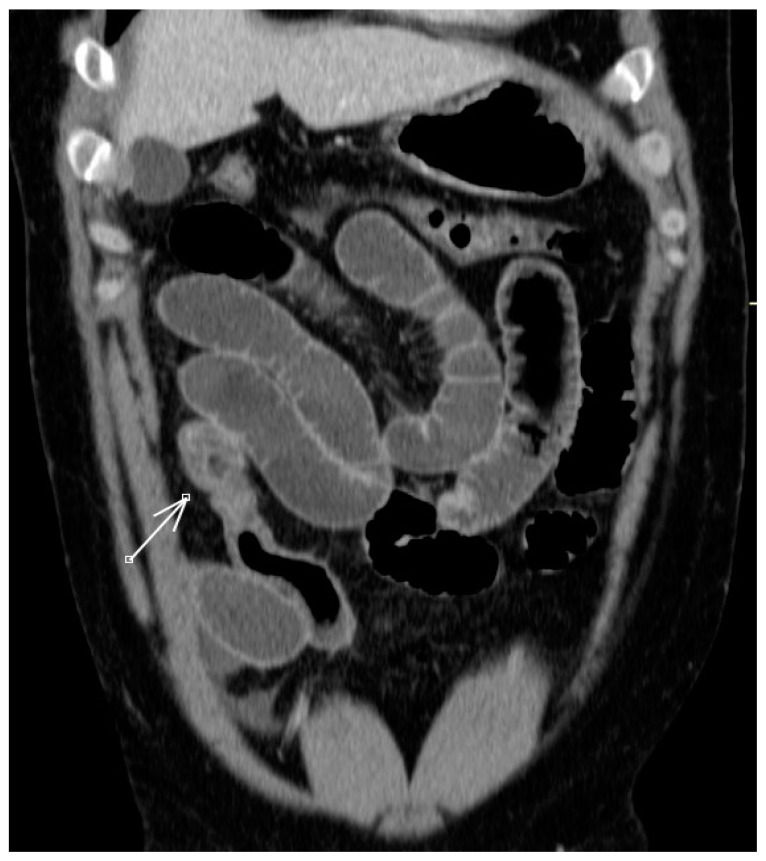
CT of abdomen coronal reconstruction: thickening of the wall of the loop of ileum, irregular morphology. The arrows represent asymmetric mural thickening of the intestinal wall with submucosal edema.

Based on these findings, a decision was made to perform an abdominal CT scan with venous phase VSD (90 mL of iodinated contrast at 2 mL/s). The scan revealed the following observations: there was asymmetric mural thickening of the intestinal wall with submucosal edema, affecting a short segment of approximately 4 cm in length in the distal ileum located in the right lower quadrant (Figure 1 and Figure 2) [18,19].

He presented a subocclusive picture with dilation of proximal bowel loops, as well as the presence of fluid and air-fluid levels inside. The patient presented a subocclusive condition characterized by dilation of the proximal bowel loops and the presence of fluid and air-fluid levels. These findings were accompanied by infiltration of the mesenteric fat, subcentimeter mesenteric lymphadenopathy, and a minimal amount of free fluid in the right parietocolic leak (Figure 1) [19,20].

**Figure 3:**
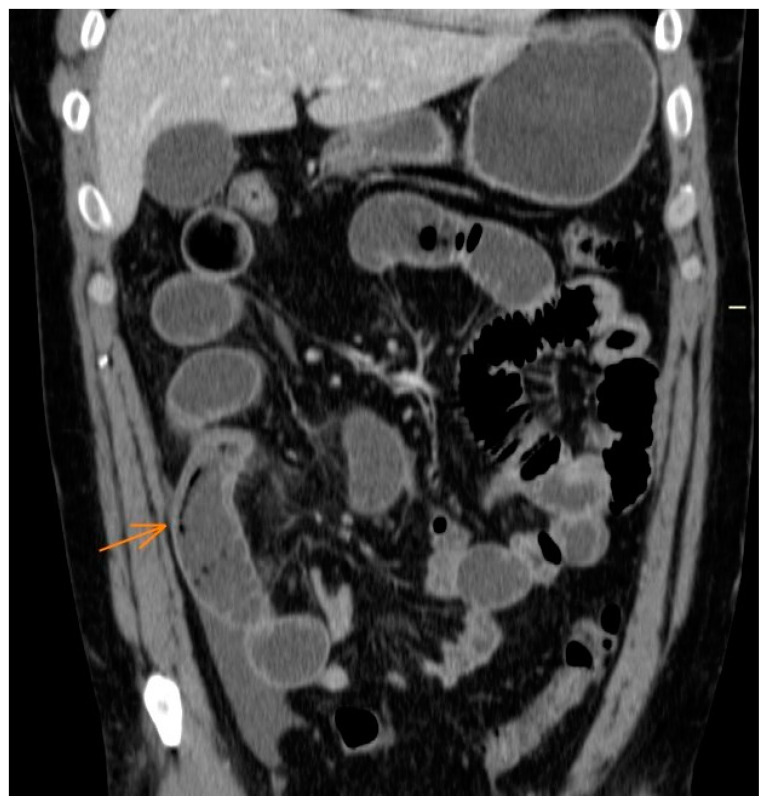
Abdominal CT: linear hypodense images in the lumen of the distal ileal loop in relation to the parasite. The arrows represent asymmetric mural thickening of the intestinal wall with submucosal edema.

The patient’s subocclusive presentation of the small intestine raised the possibility of tumor or inflammatory–infectious causes. An exploratory laparotomy was performed, which involved resecting the affected segment of the distal ileum, measuring approximately 10/15 cm in length, along with its mesentery. A mechanical side-to-side anastomosis was performed. The surgical intervention was justified by the possibility of the existence of lymphoma and, finally, due to the obstruction and ischemia of the intestinal segment [19,20]. The histological examination of the resected distal ileum revealed a dense transmural eosinophilic inflammatory infiltrate extending to the serosa, as well as the presence of a parasite in the intestinal lumen with morphological characteristics consistent with Anisakis. These histological findings prompted a re-evaluation of the CT scan, which revealed bowel obstruction and hypodense “serpiginous” linear images within the small intestine lumen, indicative of the presence of the parasite (Figure 3) [17,18,20]. 

A new medical history was obtained from the patient, who reported frequent consumption of marinated raw fish in recent months. Following the surgical intervention, a physiotherapy treatment plan was implemented, which included kinesitherapy and therapeutic physical exercises during the hospital phase. The exercise regimen was continued in the outpatient phase, with the addition of the pulsed negative pressure procedure.

The physiotherapy treatment focused on mobility exercises for the upper and lower extremities, respiratory physiotherapy, active exercises for overall mobility (such as walking and movements related to basic daily activities), and strength exercises with resistance training [21]. The pulsed negative pressure procedure, utilizing the Physium^®^ Vacuum Treatment device, specifically targeted scar tissue to prevent adhesions and myofascial retraction that could limit mobility [22]. The patient’s quality of life was assessed using the SF12 questionnaire.

## 3. Discussion

Anisakiasis is the condition in which humans are parasitized by the larvae of Anisakis simplex after consuming fish that are infected with live larvae. Anisakis simplex is a whitish helminth parasite that can reach a length of up to 3 cm [12]. The rate of fish parasitism in the fish we consume is quite high in our environment, ranging from 40% to 80%, depending on the species [12]. 

The intestinal form of anisakiasis typically manifests as acute abdominal pain accompanied by signs of peritoneal irritation, nausea, vomiting, diarrhea, and occasionally fever. The terminal ileum is the site most commonly affected. Radiological findings, in general, are non-specific, characterized by thickening of the ileum wall, which can be symmetrical or asymmetrical, along with submucosal edema. This is often accompanied by infiltration of adjacent mesenteric fat and reactive adenopathies, which may result in an occlusive condition [7,8]. Recent investigations have confirmed these imaging findings [23], highlighting the importance of an accurate differential diagnosis to guide appropriate treatment choices.

The most specific finding that could aid in the diagnosis is the identification of the parasite within the intestinal lumen, appearing as a filling defect with a characteristic “serpiginous” morphology. However, this finding is observed in only a few cases (Figure 3) [18,20]. Due to the non-specific nature of the radiological findings, the differential diagnosis is broad, including conditions such as Crohn’s disease (due to location), tumors (such as adenocarcinoma, characterized by asymmetric wall thickening), and inflammatory/infectious processes. It is important to consider the possibility that the symptoms are attributed to acute cases of anisakiasis, as reported in the literature over the past two decades [24]. 

Additionally, it is worth noting that physiotherapy treatment plays a crucial role in achieving functional recovery following such a significant surgical intervention. Early mobilization programs have been shown to facilitate recovery after abdominal surgery [21,25]. Active patient participation and targeted scar treatment can help prevent complications, similar to the benefits observed with physiotherapy intervention in the aftermath of cesarean section procedures [26].

Previous research on this subject coincides in pointing out the importance of exercise for therapeutic purposes to promote recovery after abdominal surgery [27,28]. In this sense, Boden et al. (2018) indicate that physiotherapy prevents post-surgical complications, increasing the patient’s physical capacity and reducing mortality, all of which contribute to reducing socioeconomic costs [27]. More specifically, according to Svensson-Raskh et al. (2021) [28], early mobilization promotes circulation and ventilatory mechanics, which stimulates oxygenation levels and increases functionality. The results obtained promote the inclusion of physiotherapy programs for the recovery of the patient after a surgical procedure in the abdominal region, taking as a reference the exercise and soft tissue mobilization therapies.

## 4. Conclusions

Anisakiasis has significant clinical implications, and its increasing incidence in Spain, attributed to the high consumption of fish and the recent culinary trends of consuming it undercooked or raw, necessitates considering it as a potential diagnosis for digestive diseases. Due to the non-specific radiological findings associated with the intestinal form of the disease, it is advisable to include it in the differential diagnosis of inflammatory causes, with support from a thorough medical history. Surgical intervention plays a crucial role in the management of anisakiasis, and post-surgical physiotherapy treatment promotes the patient’s short- and long-term recovery, ultimately enhancing their overall quality of life. 

## Data Availability

Not applicable.
